# Prediction and Analysis of Electrical Accidents and Risk Due to Climate Change

**DOI:** 10.3390/ijerph16162984

**Published:** 2019-08-20

**Authors:** Min-Chang Jeong, Jaehyuck Kim

**Affiliations:** Department of Electrical Engineering, Wonkwang University, Jeonbuk, Iksan 54538, Korea

**Keywords:** climate change, electrical fire, electrical accident, electric safety, risk prediction, risk rating

## Abstract

The industrial development and the increase in the use of fossil fuels have been accelerating global warming and climate change, thereby causing more frequent and intense natural disasters than ever before. Since electrical facilities are generally installed outdoors, they are greatly affected by natural disasters, thus accidents related to electrical equipment has been on the rise. In this paper, we present the risk rating associated with climate change by analyzing the statistics of electrical fires, electric shock accidents and electrical equipment accidents caused by domestic climate change. Further, we present a risk rating analysis model for electrical fires on a monthly basis through the data analysis of electrical hazards associated with various regional (metropolitan city) climatic conditions (temperature, humidity), and analyze the accident risk rating for natural disasters related to low and high voltage equipment. Through this risk analysis model for each region and type of equipment, we presented a basic prediction model for electrical hazards. Therefore, it is possible to provide electrical safety services in the future by displaying a risk prediction map of electrical hazards for each region and type of electrical equipment through web sites or smart phone apps using the presented analysis data. Further, efforts should be made to increase the robustness or reliability of electrical equipment in order to prevent electrical accidents caused by natural disasters due to climate change in advance.

## 1. Introduction

Abnormal climate phenomena appear in every corner of the world due to global warming, resulting in not only melting glaciers and rising sea levels but increasing the frequency of natural disasters such as droughts, floods, and typhoons. Abnormal climate has led to a situation where human life as well as the ecosystem is threatened. Because of global warming triggered by the increased use of fossil fuels with the industrial development, the climate is changing the environment such that extremely cold and hot temperatures are deepened and the frequency and intensity of lightning and typhoons are increased, which adversely affects electrical equipment [1,2,3]. Abnormal climate phenomena such as sea level rise and changes in temperature and precipitation patterns due to continuous climate change are increasingly affecting various living environments and systems such as ecosystems, transportation, energy supply and demand, and infrastructure [4,5].

In order to improve the predictability of electrical hazards, it is necessary to develop technologies that can respond to environmental changes by identifying, evaluating, and analyzing the risks through the analysis of the vulnerability of electrical equipment according to climate change [6].

In this paper, we statistically analyze the impacts of electrical accidents (fire, failure, etc.) in South Korea, arising from global warming manifested in abnormal climate conditions such as high temperatures and localized heavy falls, on electrical equipment, and then suggest and predict the risk rating of electrical equipment based on this analysis.

## 2. Statistics on the Causes of Electrical Equipment Accidents Due to Climate Changes

Damage to electrical equipment due to domestic climate change such as heavy rains, floods, typhoons, lightning, icy snow and salt damage has been gradually increasing equipment failures or more frequent fire occurrence. Further, electricity consumption due to the increased use of cooling and heating systems caused by severe hot and cold weather has been growing. These various climate changes affect electrical equipment and the durability of electrical equipment to natural disasters is essential to reducing damage.

Temperature rise due to continuous global warming causes transformer explosions and affects electrical equipment, and the increase in frequency and intensity of typhoons and the inundation of underground electrical equipment because of melting glaciers and rising sea level are expected to increase salt damage [7,8]. The number and component ratio of electrical equipment accidents caused by various climate phenomena become more diverse.

Table 1 shows the statistics on the number of electrical equipment accidents caused by floods from 2006 to 2015. Table 2 shows the number of electrical equipment accidents and the component ratio for total accidents caused by wind (gale, typhoon) and icy snow (heavy rain, heavy snow) from 2006 to 2015. Equipment accidents caused by natural disasters were decreasing a little bit up until 2010 but they had sharply surged since 2010. They were decreasing again until 2015 but have been increasing up until now since 2015. Table 3 shows the number of annual electrical equipment accidents caused by dust and salt damage from 2006 to 2015. Equipment accidents were decreasing gradually between 2006 and 2015 except sudden increase in 2009 and 2012. Natural disasters caused by abnormal climate have been gradually increasing or intensifying, resulting in more damage to electrical equipment in the future. Improving the robustness of individual equipment vulnerable to natural disasters is a very pressing issue.

## 3. Correlation Analysis between Electrical Fires and Various Climate Variables

**Figure 1:**
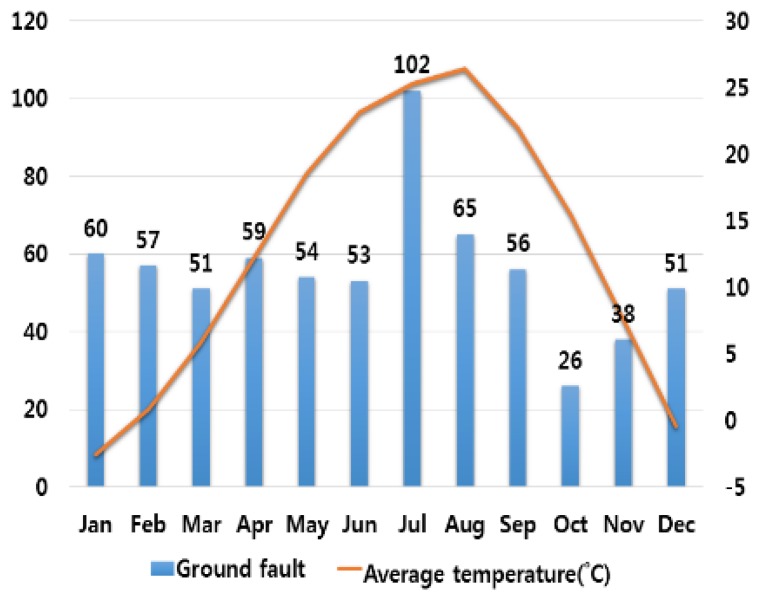
Correlation between the number of fires caused by ground fault and the average temperature on a monthly basis.

**Figure 2:**
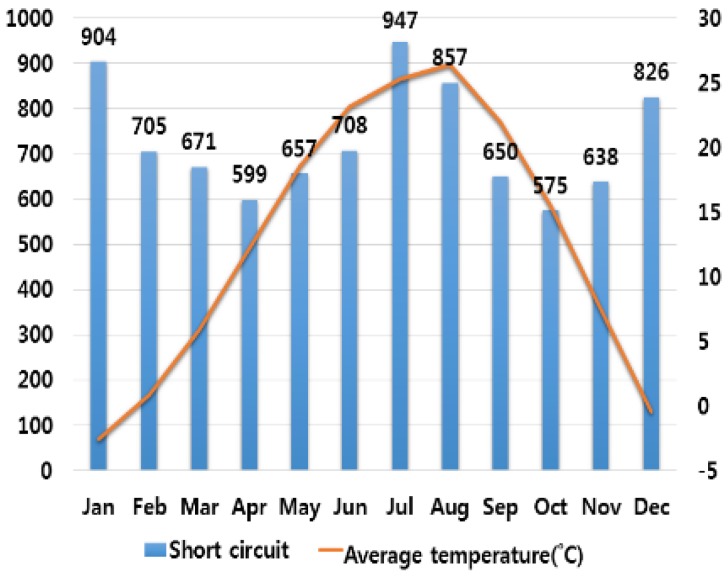
Correlation between the number of fires caused by short circuit and the average temperature on a monthly basis.

**Figure 3:**
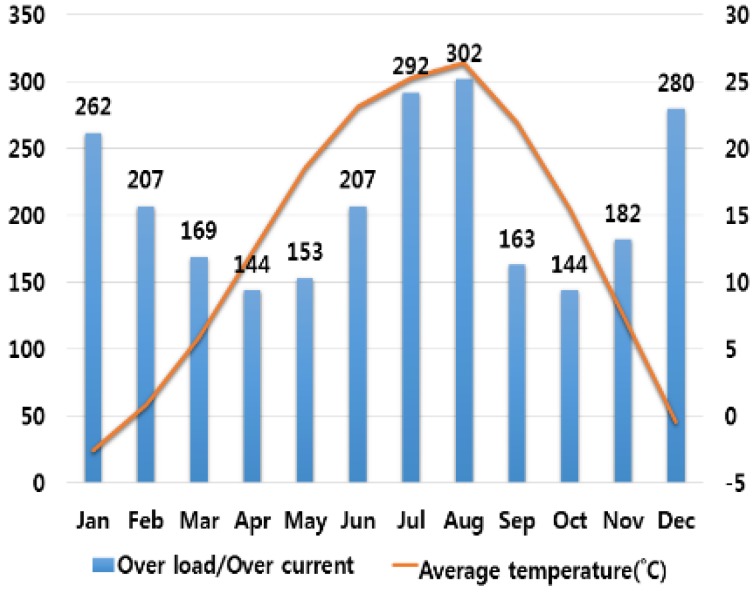
Correlation between the number of fires caused by overload/over current and the average temperature on a monthly basis.

**Figure 4:**
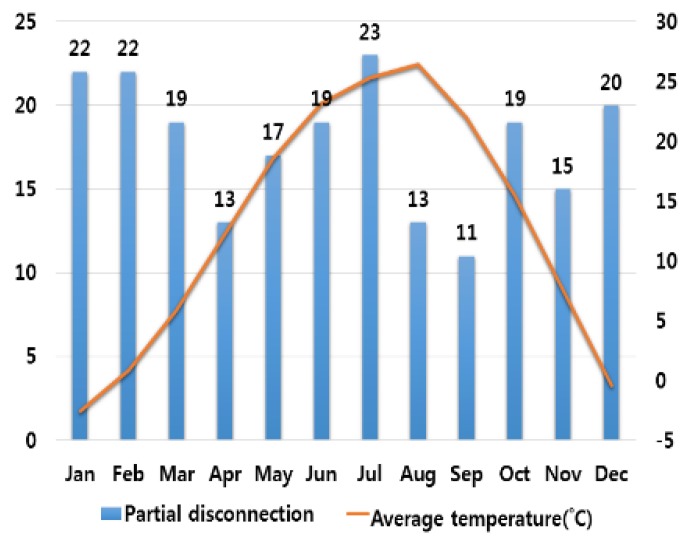
Correlation between the number of fires caused by partial disconnection and the average temperature on a monthly basis.

**Figure 5:**
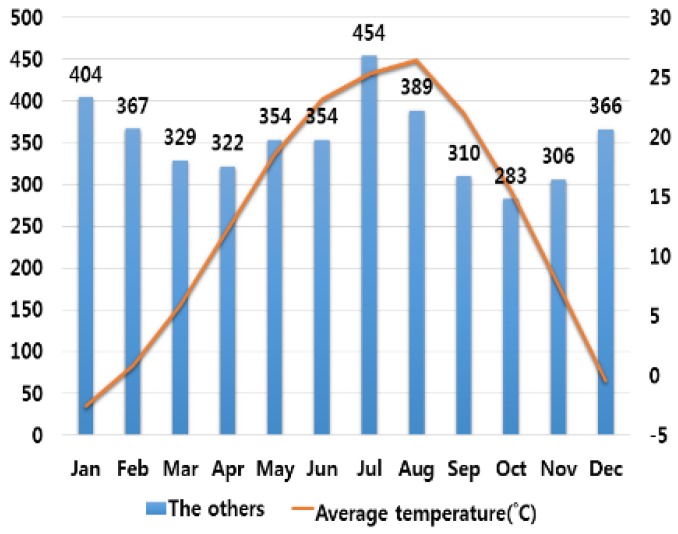
Correlation between the number of fires caused by other causes and the average temperature on a monthly basis.

**Figure 6:**
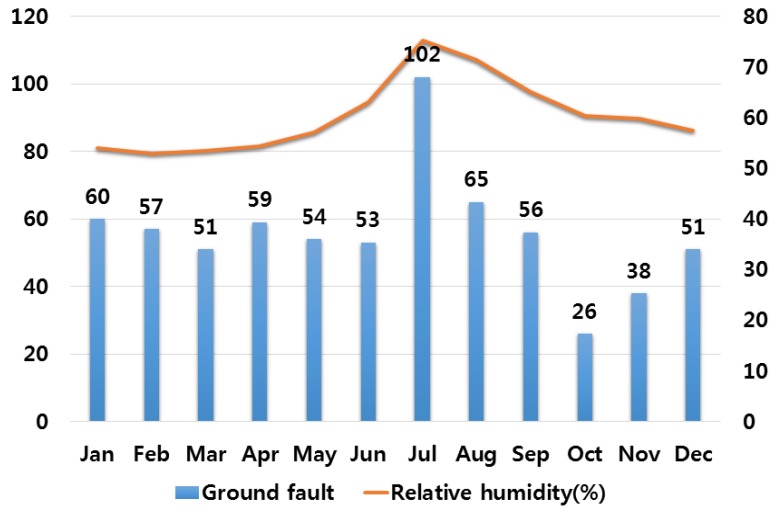
Correlation between the number of fires caused by ground fault and the average humidity on a monthly basis.

**Figure 7:**
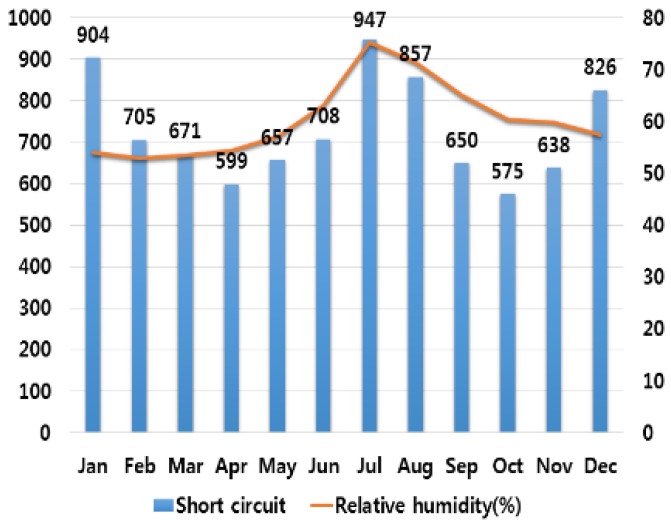
Correlation between the number of fires caused by short circuit and the average humidity on a monthly basis.

**Figure 8:**
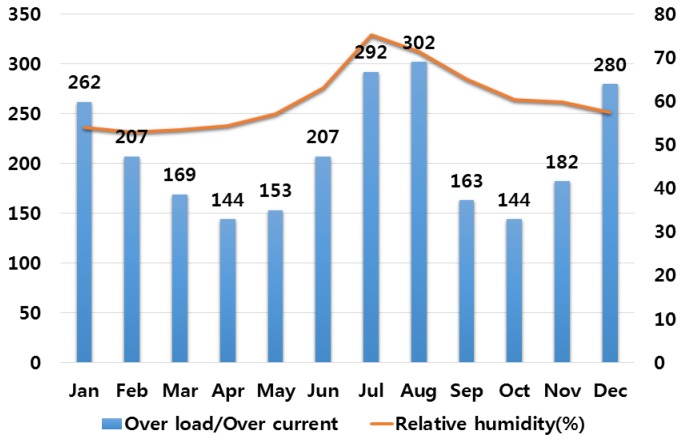
Correlation between the number of fires caused by overload/over current and the average humidity on a monthly basis.

**Figure 9:**
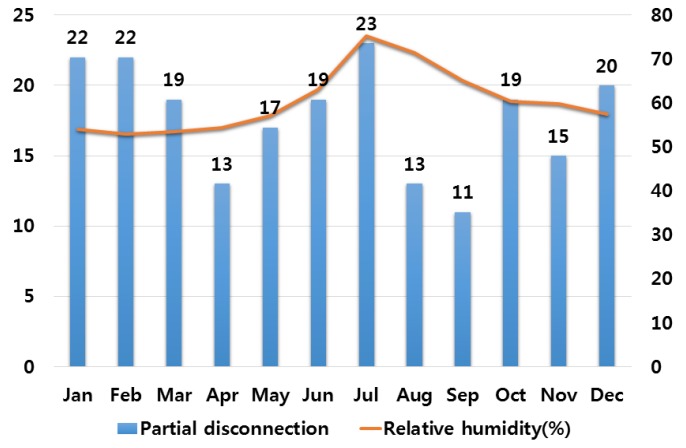
Correlation between the number of fires caused by partial disconnection and the average humidity on a monthly basis.

**Figure 10:**
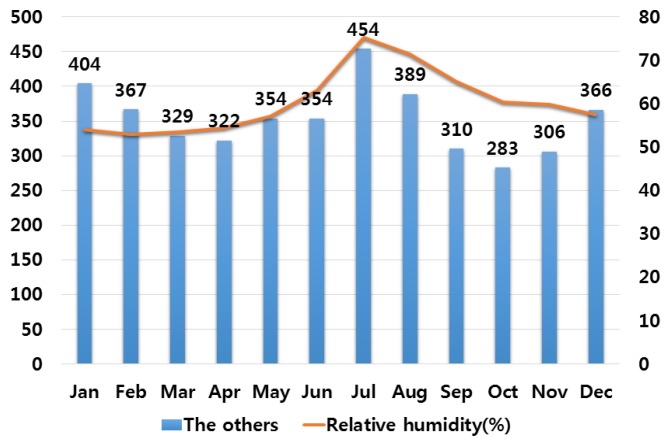
Correlation between the number of fires caused by other causes and the average humidity on a monthly basis.

In order to analyze the correlation between temperature and humidity, which are typical climate variables, and electrical fires, we investigated monthly average temperature and humidity and the number of monthly electrical fires for ten major cities in Korea. Electrical fires are classified according to the causes of ignition. Among ten cities, only the result of investigation on Seoul are presented in this paper due to lack of space. Figure 1, Figure 2, Figure 3, Figure 4 and Figure 5 respectively shows the correlation between the monthly average temperature of and the number of monthly electrical fires of Seoul in regards to the five causes of ignition. Figure 6, Figure 7, Figure 8, Figure 9 and Figure 10 respectively shows the correlation between the monthly average humidity of and the number of monthly electric fires of Seoul in regards to five causes of ignition. The charts shown in Figure 1, Figure 2, Figure 3, Figure 4, Figure 5, Figure 6, Figure 7, Figure 8, Figure 9 and Figure 10 are all based on the statistical data of Korea Meteorological Administration [10] and Korea Electric Safety Corporation and Korea Electrical Safety Corporation (KESCO) [11].

Overall, fires occurred most frequently in July and August when temperature and humidity were highest, and in December and January when temperature and humidity were lowest. This is attributed to the fact that the use of cooling and heating systems and electric heaters is concentrated during this period. Most causes of fires are concentrated in summer and winter but the number of fires caused by short circuit/ground fault is conspicuously high only in summer (July). Lightning strikes occur primarily in summer and are considered to cause a ground fault or line-to-line short circuits by destroying the insulation of the transmission lines. For partial disconnection, the number of fires is more concentrated in cold winter (December to February) than in hot summer. This is attributable to the surge in demand for electric heating systems in winter. Overall, humidity also shows a similar tendency to temperature.

## 4. Risk Rating for Electrical Fires by Region According to the Cause of Electrical Fires

The number of electrical fires per 100,000 people in each region according to five different causes of electrical fires was analyzed as shown in Table 4. These data are based on ‘Statistical Analysis on the Electrical Accidents’ by Korea Electrical Safety Corporation. From Table 4, we made a table for a relative risk rating (Table 5) by setting the maximum value as ‘very high’, the minimum value as ‘very low’, and the middle value as ‘moderate’. Based on the risk rating in Table 5, the risk rating for electrical fires by cause for each region can be represented as in Table 6.

There are fewer electrical fires in Seoul and Busan despite huge populations. In particular, there are lots of fires in Gangwon (Chuncheon city) relative to population size, which might be affected by the lack of disaster prevention infrastructure such as firefighting facilities. As shown in Table 6, there are four cities (Incheon, Daejeon, Chuncheon, and Jeju) with at least two ratings higher than ‘high’, and five cities (Daejeon, Gwangju, Cheongju, Chuncheon, and Jeju) with at least one ‘very high’ rating. Busan is the only city with no ‘high’ or ‘very high’ rating. While Daegu has the lowest average risk rating, Chuncheon has the highest average risk rating.

**Figure 11:**
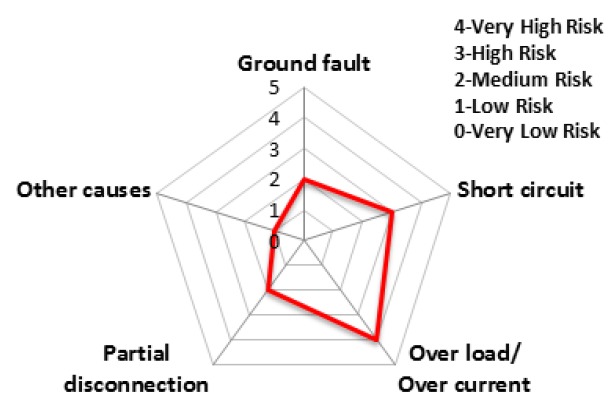
The risk rating for electrical fires in Seoul.

**Figure 12:**
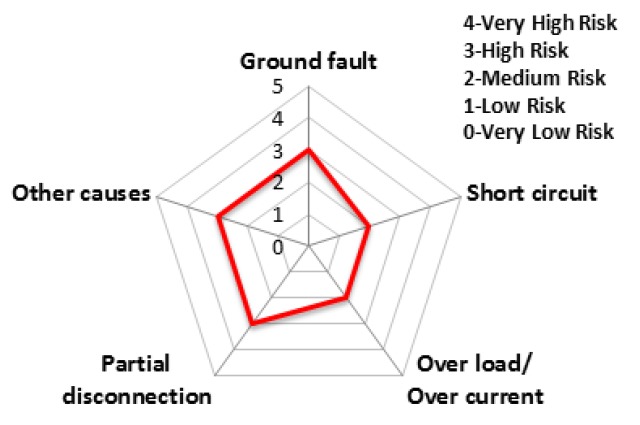
The risk rating for electrical fires in Busan.

**Figure 13:**
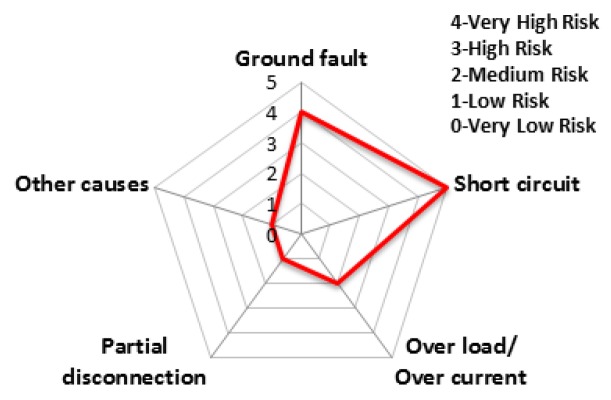
The risk rating for electrical fires in Daegu.

**Figure 14:**
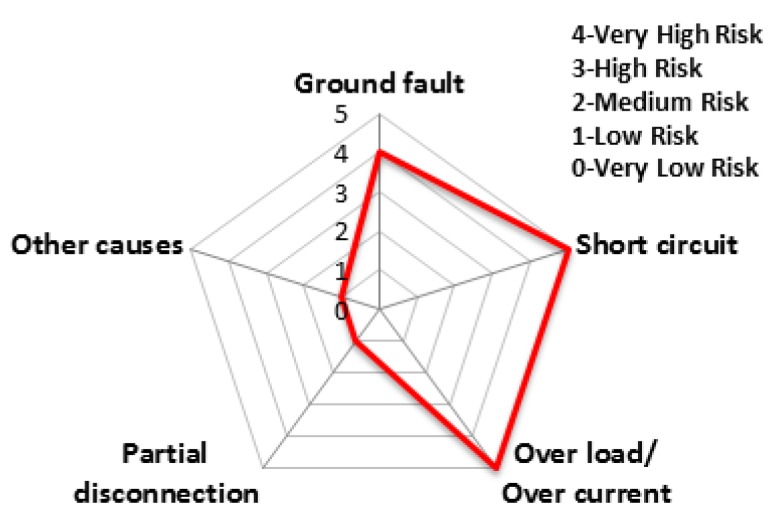
The risk rating for electrical fires in Daejeon.

**Figure 15:**
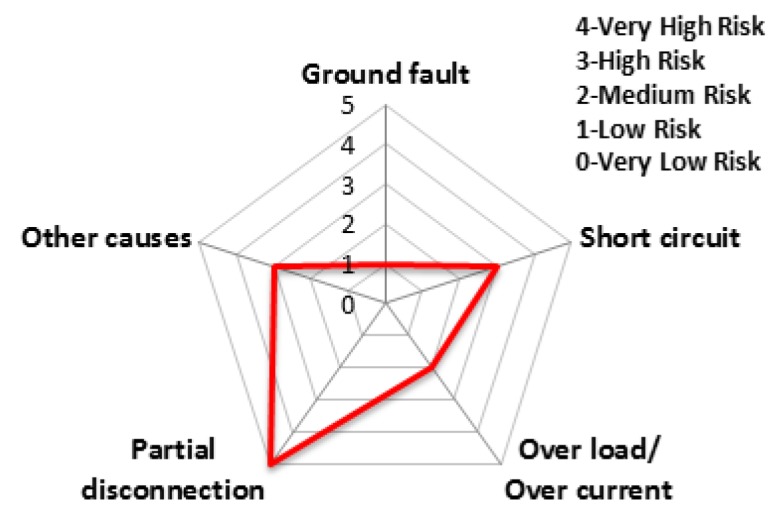
The risk rating for electrical fires in Gwangju.

**Figure 16:**
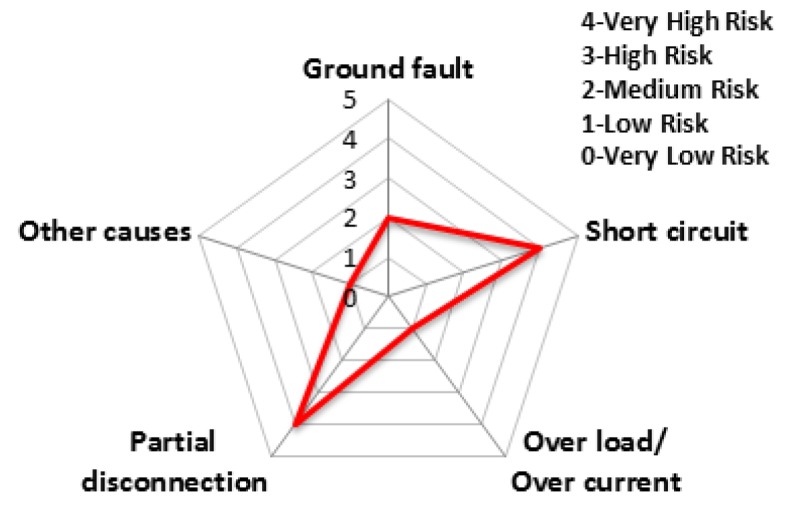
The risk rating for electrical fires in Incheon.

The charts from Figure 11, Figure 12, Figure 13, Figure 14, Figure 15 and Figure 16 represent the risk rating by cause from Table 6 using a spider web chart. There are five different categories of causes for electrical fires. Each region has different causes of the highest and lowest risk rating. Seoul and Busan have relatively low risk rating comparing other regions. Daejeon has three factors with the rating higher than level 4 (very high risk) while Busan has no factors with higher than level 4.

**Figure 17:**
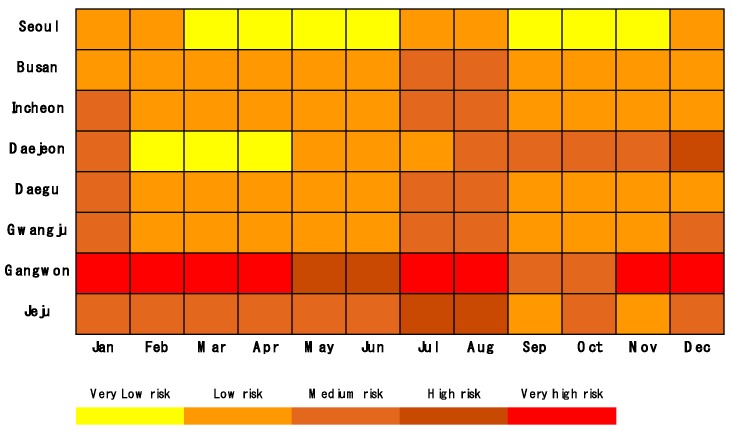
Risk matrix diagram for electrical fires by region and month.

So far, we have analyzed the annual electrical fires in each region and correlated them with monthly climate change variables. In addition, annual fires by cause in each region are analyzed. By integrating electric fires by region into the monthly climate variables, accumulated for 10 years (from 2006 to 2015) of statistics, the average number of electrical fires in each region in each month can be represented. Figure 17 shows the monthly electrical fires by region in the form of matrix diagram [12] that indicates the correlation between the climate variables and the electrical fires. This diagram shows the risk level of electrical fires in any month of the year in each region. The redder the color in the matrix, the higher the risk is. On the other hand, the more yellow the color is, the lower the risk is.

If this matrix is made based on 10 years (from 2011 to 2019) of statistical data, it can be predicted that the risk level of any area in any month of the next year after that period will be similar to that of this matrix. For example, in 2020, most regions are predicted to have a high fire risk level from July to August and December to January. The risk level for electrical fires in Gangwon is likely to be particularly high through the entire year compared to other regions.

## 5. Risk Rating for Electrical Accidents by Electrical Equipment

As climate change accelerates, more frequent disasters are occurring. Electrical equipment is often installed outdoors, and if such electrical equipment is affected by natural disasters, they can have a significant impact on society. Therefore, efforts are needed to increase the resistance of electrical equipment to natural disasters that can be caused by climate change. Accidents in electrical equipment result from various internal and external causes. However, since the electrical equipment is mainly installed outside, it is affected by various climatic conditions (heat, heavy rain, heavy snow, etc.). Even in the case of the indoor equipment, it cannot be considered to be independent of climate various due to the abrupt load change and interlocking with outdoor facility.

**Figure 18:**
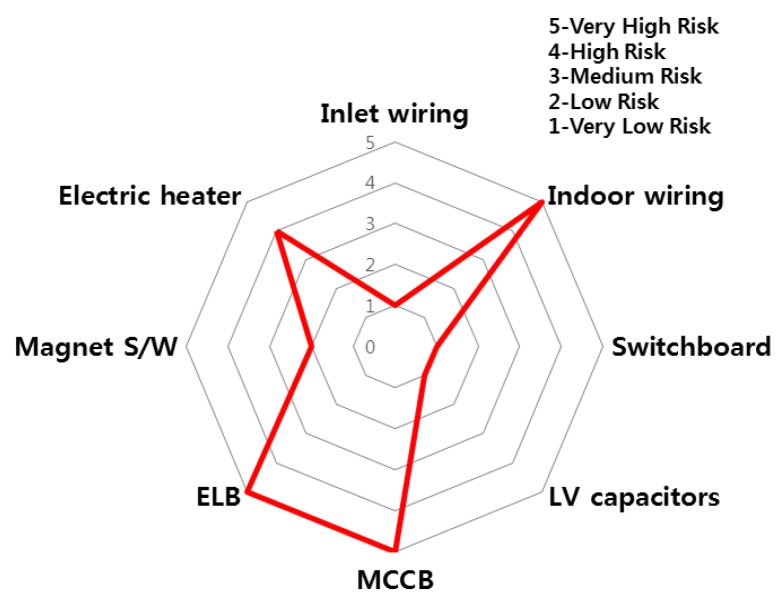
The risk rating for low voltage equipment by device.

**Figure 19:**
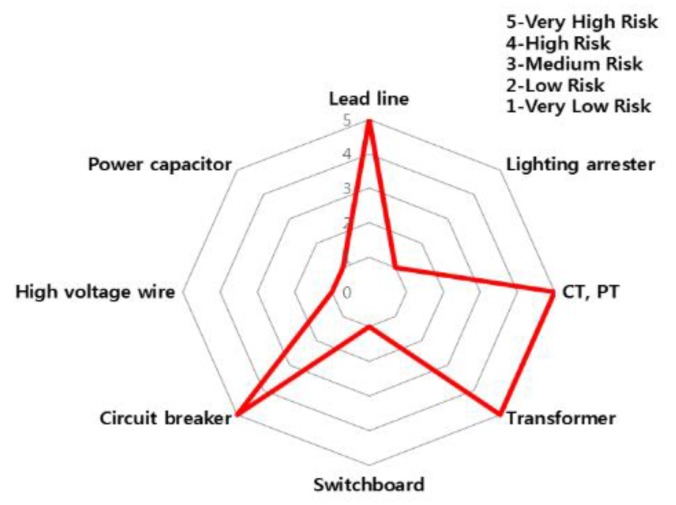
The risk rating for high voltage equipment by device.

**Figure 20:**
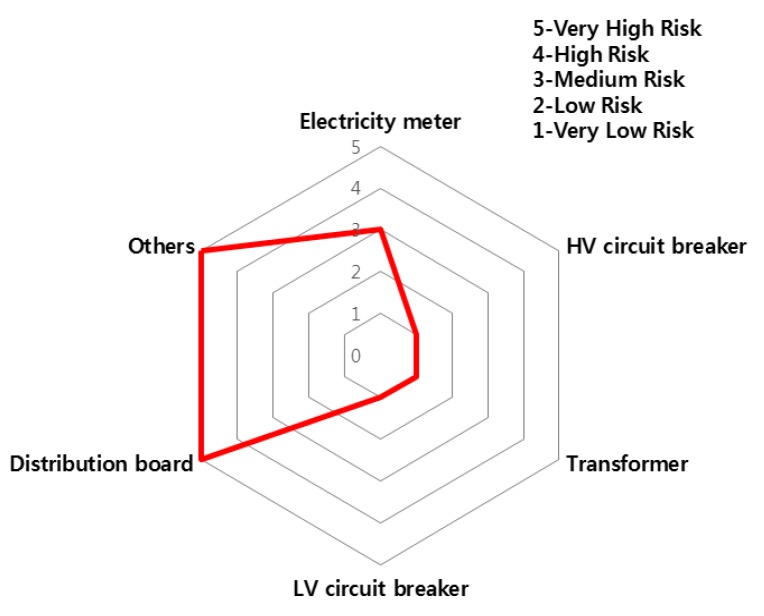
The risk rating for ignition equipment by device.

The accident statistics of electrical equipment according to the monthly climate variations, the risk rating and monthly risk prediction of each equipment are proposed in this section. The risk rating for equipment accidents was specified using the number of accidents by low/high voltage electrical equipment and ignition equipment. Table 7 shows the risk rating for the number of accidents by low (110~440 V) and high voltage (3.3~22.9 kV) equipment and ignition equipment. Based on this table, the accident risk rating for electrical equipment is shown in Table 8 and Table 9 and Figure 18 and Figure 19 in the form of a table and a spider web chart. The accident risk rating for ignition equipment is shown in Table 10 and Figure 20. These data are based on ‘Statistical Analysis on the Electrical Accidents’ by Korea Electrical Safety Corporation [13].

Indoor wiring has the highest accident risk rating for low voltage equipment and the transformer has the highest accident risk rating for high voltage equipment. Distribution board has the highest accident risk rating for electrical fires for ignition equipment.

**Figure 21:**
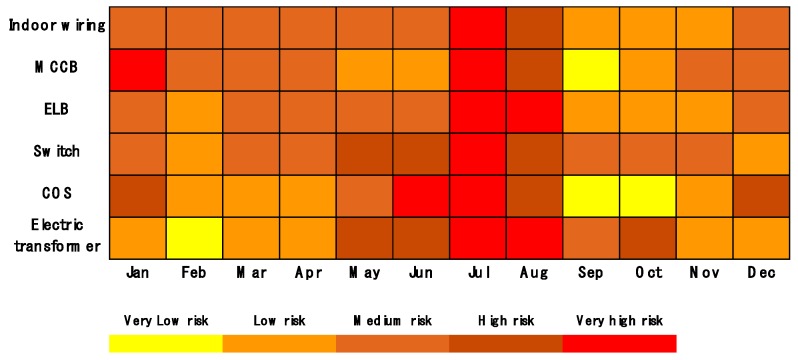
Risk matrix diagram of the electrical fires by equipment and month.

Figure 21 shows an electrical fire risk matrix indicating the prediction of the risk level for electrical fires by each type of equipment on a monthly basis. Similar to Figure 17, the redder the color in the matrix, the higher the risk is. On the other hand, the more yellow the color is, the lower the risk is. The accident rate of all types of equipment is high in July and MCCB accidents happen frequently in January compared to other types of equipment. With the 10 years of data, in addition to the annual update, the accuracy of this matrix model will increase.

## 6. Conclusions

Korea is one of the countries with the greatest levels of climate change. In this regard, it is necessary to study the statistical analysis of electrical accidents and fires caused by recent rapid climate changes. Besides, study of risk ratings and risk forecasting for each region, cause, and electrical equipment is also urgently needed.

This paper describes a statistical relationship between climate variables and electrical accidents and fires in Korea. In this regard, the risk rating by region, cause and equipment is presented. In addition, a risk matrix for monthly fire risk prediction by region as well as risk matrix for predicting the monthly accident risk prediction by type of equipment are presented.

We first presented a statistical analysis of electrical fires on a monthly basis associated with various regional (metropolitan city) climatic conditions (temperature, humidity). Next, we presented an electrical fire risk rating for each different region and each type of equipment. Based on the risk rating analysis for each region and type of equipment, we presented risk matrix diagrams to visualize the likelihood of electrical fires by each region in any month of the year or the likelihood of electrical accidents by each type of electric equipment in any month of the year. Since this matrix diagram is based on 10 years of statistical data, it is possible to forecast approximately the risk level of electrical fires or accidents beyond that period. Such a prediction will be more accurate as the data accumulates annually accumulates.

Meanwhile, we are going to propose a method of displaying the risk level prediction map of electrical hazards for each region and type of electrical equipment through web sites or smartphone apps in the future based on using the analysis data presented in this study.

## Figures and Tables

**Table 1:** The number of annual electrical equipment accidents caused by floods.

Year	No. of Accidents	Ratio (%)
2006	399	6.7
2007	494	7.1
2008	319	5.6
2009	361	4.2
2010	464	5.6
2011	571	7.3
2012	451	5.3
2013	415	5.6
2014	273	4.3
2015	170	2.9

Source: Fire Statistic, National Fire Protection Information Center [9].

**Table 2:** The number of annual electrical equipment accidents caused by wind damage, heavy snowfall, cold wave, and freezing.

Year	No. of Accidents	Ratio (%)
2006	180	3
2007	260	3.7
2008	262	4.6
2009	149	1.7
2010	214	2.6
2011	424	5.4
2012	476	5.6
2013	150	2
2014	158	2.5
2015	80	1.4

Source: Fire Statistic, National Fire Protection Information Center [9].

**Table 3:** The number of annual electrical equipment accidents caused by dust and salt damage.

Year	No. of Accidents	Ratio (%)
2006	74	1.2
2007	57	0.8
2008	47	0.8
2009	62	0.7
2010	55	0.7
2011	45	0.6
2012	62	0.7
2013	50	0.7
2014	47	0.7
2015	33	0.6

Source: Fire Statistic, National Fire Protection Information Center [9].

**Table 4:** The number of electrical fires by metropolitan city and city per 100,000 people.

Region	Number of Electrical Fires by Cause				
	Ground Fault	Short Circuit	Overload	Partial Disconnection	Others
Seoul	68	880	252	21	427
Gwangju	49	991	182	36	621
Daegu	53	726	165	11	825
Daejeon	88	1296	186	17	428
Busan	80	764	180	25	640
Jeju	50	1300	295	26	477
Cheongju	116	526	214	7	605
Jeonju	78	807	181	8	732
Chunchein	110	1268	413	64	1411
Incheon	59	1073	145	29	433
Min./Max.	49/116	526/1300	145/413	7/64	427/1411
Avg.	75	963	221	24	660

Source: “Statistical Analysis of Electric Disaster” by Korea Electrical Safety Corporation (KESCO).

**Table 5:** The risk rating scale for electrical fires by cause.

Rating	Ground Fault	Short Circuit	Overload	Partial Disconnection	Others
Very low (1)	0~53	0~673	0~155	0~17	0~462
Low (2)	54~68	674~867	156~199	18~22	463~594
Moderate (3)	69~84	868~1060	200~243	23~26	595~726
High (4)	85~98	1061~1252	244~287	27~31	727~858
Very high (5)	99>	1253>	288>	32>	859>

Source: “Statistical Analysis of Electric Disaster” by Korea Electrical Safety Corporation (KESCO).

**Table 6:** The risk rating for electrical fires by region.

Region	Risk Rating of Electrical Fires				
	Ground Fault	Short Circuit	Overload	Partial Disconnection	Others
Seoul	2	3	4	2	1
Busan	3	2	2	3	3
Incheon	2	4	1	4	1
Daejeon	4	5	2	1	1
Daegu	1	2	2	1	4
Gwangju	1	3	2	5	3
Cheongju	5	1	3	1	3
Jeonju	3	2	3	1	4
Chuncheon	5	5	5	5	5
Jeju	1	5	5	3	2

Source: “Statistical Analysis of Electric Disaster” by Korea Electrical Safety Corporation (KESCO).

**Table 7:** The risk rating scale for electrical equipment.

Rating	Low Voltage Equipment (110~440 V)	High Voltage Equipment (3.3~22.9 kV)	Ignited Equipment
Very low (1)	~170	~47	~87
Low (2)	171~219	48~61	88~113
Moderate (3)	220~268	62~76	114~139
High (4)	269~317	77~90	140~164
Very high (5)	318~	91~	165~

**Table 8:** The number of accidents and risk rating for low voltage equipment.

Device	No. of Accidents	Rating
Inlet wiring	24	1
Indoor wiring	505	5
Switchboard	107	1
LV capacitors	49	1
MCCB	322	5
ELB	456	5
Magnet S/W	188	2
Electric heater	302	4

LV: Low voltage; MCCB: Mold case circuit breaker; ELB: Electric leakage breaker; S/W: Switch.

**Table 9:** The number of accidents and risk rating for high voltage equipment.

Device	No. of Accidents	Rating
Lead line	103	5
Lighting arrester	36	1
CT, PT	106	5
Transformer	140	5
Switchboard	30	1
Circuit breaker	115	5
High voltage wire	16	1
Power capacitor	3	1

CT: Current transformer; PT: Potential transformer.

**Table 10:** The number of accidents and risk rating for ignition equipment.

Device	No. of Accidents	Rating
Electricity meter	123	3
HV circuit breaker	13	1
Transformer	49	1
LV circuit breaker	20	1
Distribution board	366	5
Others	187	5
High voltage wire	16	1
Power capacitor	3	1

HV: High voltage.
